# Nextpie: a web-based reporting tool and database for reproducible nextflow pipelines

**DOI:** 10.1093/bioadv/vbaf252

**Published:** 2025-10-10

**Authors:** Bishwa Ghimire, Nicholas Booth, Tapio Lönnberg, Tero Aittokallio

**Affiliations:** Medicity Research Laboratory, University of Turku, Turku, 20520, Finland; Institute for Molecular Medicine Finland (FIMM), Helsinki Institute of Life Science (HiLIFE), University of Helsinki, Helsinki, 00014, Finland; InFLAMES Research Flagship Center, University of Turku, Turku, 20520, Finland; InFLAMES Research Flagship Center, University of Turku, Turku, 20520, Finland; Turku Bioscience Centre, University of Turku and Åbo Akademi University, Turku, 20520, Finland; InFLAMES Research Flagship Center, University of Turku, Turku, 20520, Finland; Turku Bioscience Centre, University of Turku and Åbo Akademi University, Turku, 20520, Finland; Institute for Molecular Medicine Finland (FIMM), Helsinki Institute of Life Science (HiLIFE), University of Helsinki, Helsinki, 00014, Finland; InFLAMES Research Flagship Center, University of Turku, Turku, 20520, Finland; Oslo Centre for Biostatistics and Epidemiology (OCBE), Faculty of Medicine, University of Oslo, Oslo, 0372, Norway; Institute for Cancer Research, Department of Cancer Genetics, Oslo University Hospital, Oslo, 0424, Norway

## Abstract

**Motivation:**

High-throughput genomic data analysis consists of the inexorably intertwined inputs and outputs of a vast array of bioinformatic analysis tools. To guarantee streamlined and reproducible analyses, the often complex data analysis pipelines need to be run using workflow management tools. Nextflow is one popular tool commonly used to automate such pipelines. Nextflow records key pipeline data, such as the submission time, start time, completion time, CPU usage, memory usage, and disk usage for each task run. These data are stored in log files, often scattered across a file system. Therefore, aggregating information about resource usage critical for the optimization of Nextflow pipelines and improving reproducibility, as well as parsing and managing such log data, can quickly become cumbersome.

**Results:**

Here, we present a web-based tool, Nextpie, which provides both a database and a reporting tool for Nextflow pipelines. Nextpie stores comprehensive resource usage information in a relational database, thus facilitating and accelerating the performance of a variety of data analyses and interactive visualizations, providing an easily comprehensible overview of a pipeline’s resource usage.

**Availability and implementation:**

The Nextpie source code, user documentation, an SQLite database with test data, and a Nextflow example pipeline are available at GitHub (https://github.com/bishwaG/Nextpie).

## 1 Introduction

The popularity of high-throughput sequencing technology has contributed to the generation of enormous quantities of genomic data (Kodama *et al.* 2012). The volume of data produced by sequencing centers is increasing with every passing year as ever more life science researchers begin to incorporate sequencing data into their research. Sequencing facilities and bioinformatics core units routinely process these data for quality control, preprocessing and downstream data analyses. A recent rapid expansion in available tools has resulted in an increasingly complex and heterogeneous ecosystem of methods, file formats and dependencies. Managing such complexities manually is a challenging task. Thus, workflow management tools such as Nextflow (Di Tommaso *et al.* 2017), CWL (Crusoe *et al.* 2022), GWL (Vallet *et al.* 2022), Galaxy (The Galaxy Community 2022), Snakemake (Mölder *et al.* 2021), and Targets (Landau 2021), are becoming increasingly popular to automate data analysis pipelines and guarantee the reproducibility of workflows.

One popular workflow management tool, Nextflow, solves many problems that arise from manually managing a data analysis pipeline; however, some functionalities could be expanded upon in order to further streamline analyses. For instance, each Nextflow pipeline run can record metadata such as running time, exit status, CPU usage, memory usage, and disk I/O (Supplementary Table S1). Nextflow stores the metadata in an output folder of each pipeline run. Thus, for example after 50 pipeline runs, metadata log files will have been stored in 50 different locations in a file system. Real-time and accurate monitoring of the performance of a pipeline from such scattered data can become an insurmountable task. Sequencing centers and bioinformatics core units regularly use Nextflow-based pipelines and are not only interested in the pipeline outputs, but also in the pipeline’s resource usage and performance. Optimal use of computing resources can give them an advantage in offering competitive pricing to their customers.

**Figure 1.**
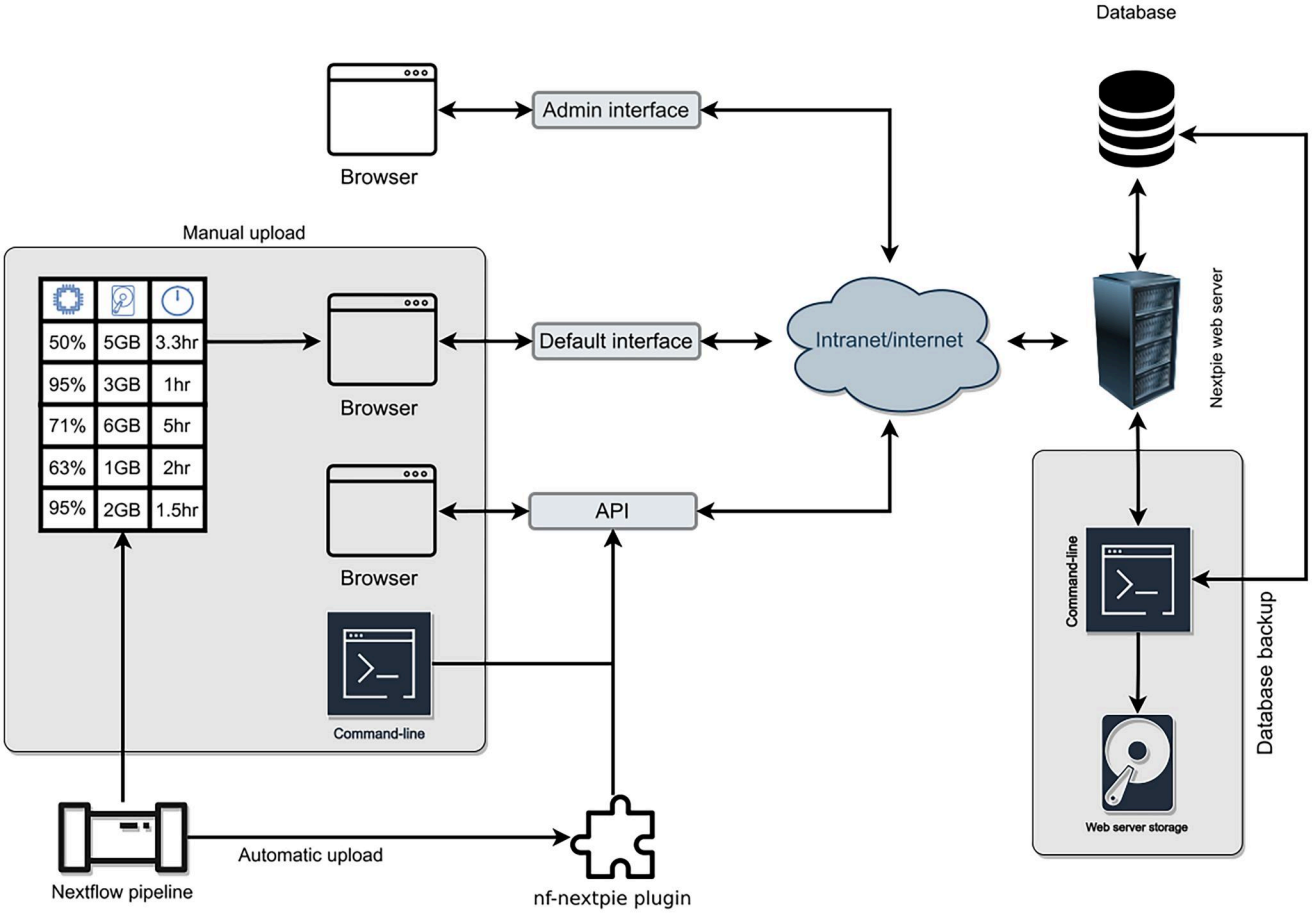
A schematic data flow diagram of Nextpie. The table in the Manual upload box represents the computational resource usage data generated by a Nextflow pipeline. This data can be uploaded manually to a relational database via Nextpie’s interfaces, such as the web-interface, command line interface, or the API. A Nextflow pipeline can upload the data automatically to Nextpie using nf-nextpie plugin on an ad hoc basis. Nextpie comes with the admin interface, the default interface, and the API for programmatic access. There is the possibility to back up the relational database automatically.

**Figure 2.**
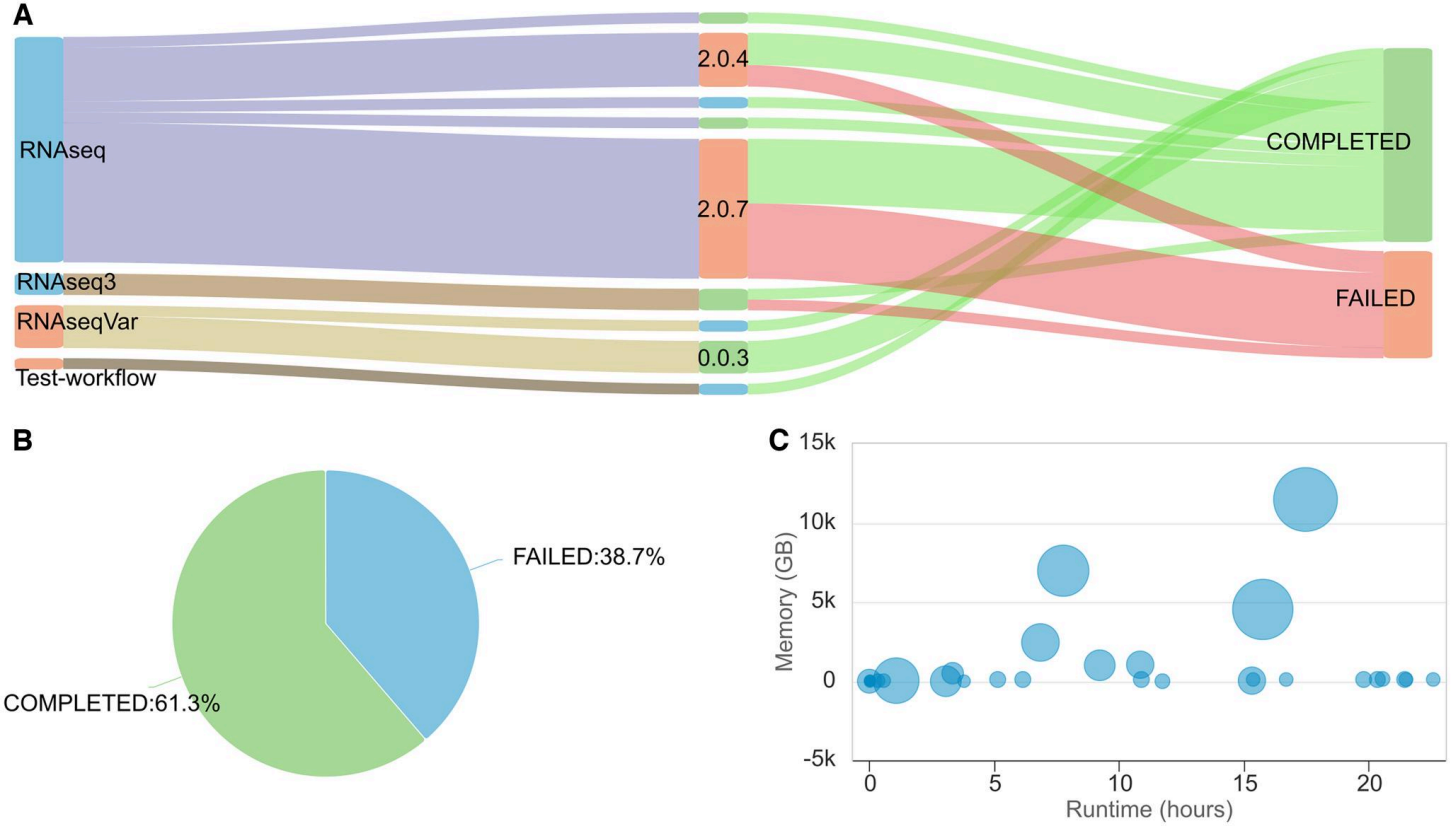
Example plots generated by Nextpie using test data. These interactive plots disclose additional details upon a mouse hover whilst rendered in Nextpie’s web-interface. (A) The alluvial plot displays failed (red segments) and completed (green segments) pipeline runs by pipeline version. On the left: pipeline names; at the middle: pipeline versions; and on the right: pipeline status. The width of the segments between any two nodes represents the number of pipeline runs. (B) The pie chart illustrates the percentage of failed and successful pipeline runs. (C) The scatter plot displays run-time versus memory consumption of all the pipeline runs. Each bubble represents a pipeline run, and its size the number of analysis processes in the run.

Here, we present the web-based tool, named Nextpie (Fig. 1), which collects (via RESTful API) resource usage metadata automatically from any Nextflow pipeline and, once properly integrated, stores these pipeline metadata in an in-house database, aggregates them, and generates interactive visualizations (Fig. 2). Nextpie therefore provides valuable insights extracted from data that would otherwise be sitting idle in a file system.

## 2 Methods

Nextpie is a web-application written in Python using the Flask framework (https://flask.palletsprojects.com). Being a web-application, it can be run as a container using Docker (Merkel 2014) or as a web-service using WSGI (Web Server Gateway Interface) servers such as Gunicorn and Waitress. WSGI servers are flexible and simple to deploy. However, for production use, we recommend running Nextpie on an in-house high-performance web-server such as Apache (Fielding and Kaiser 1997) using a reverse proxy. Apache offers better performance and security features compared to WSGi servers. The Highcharts (https://www.highcharts.com) javascript library is used to generate interactive plots in Nextpie. The plots can be exported as PNG, JPEG, PDF, or SVG files. The plot data can be exported as CSV or XLS files. The exported data allows for the use of third-party software to reproduce a plot if desired. This gives users more freedom to customize their visualizations. Another JavaScript library, Ag-grid (https://www.ag-grid.com), is used in Nextpie to browse database records. These records can be exported to a CSV file. Third party analysis software such as R can be used to generate a variety of plots using this exported data. Ag-grid library also allows the user to generate custom plots, as well as group and aggregate columns for basic analysis using Nextpie’s interface.

An SQLite3 database is used by default to store the users’ login information and computational resource usage information (Supplementary Table S1) from Nextflow pipelines. Nextpie processes the information stored in the database and can display results via its web-interface, or via an API (Application Programming Interface). Nextpie also supports more advanced relational database systems, such as PostgreSQL, for production level deployment. Python’s Flask-RESTX (https://flask-restx.readthedocs.io) RESTful API library is used to create an API endpoint and to facilitate asynchronous communication with Nextpie. The API endpoint contains Swagger API documentation with comprehensive details on each analysis task’s inputs and outputs. Authentication is performed using an API key generated via Nextpie’s web-interface. The authentication used in Nextpie is the Swagger’s API key authentication (https://swagger.io/docs/specification/2-0/authentication/).

## 3 Results

### 3.1 Input data

Nextflow’s command-line interface has the optional flag -with-trace, which generates an execution tracing file trace.txt for a single pipeline run. Alternatively, Nextflow’s configuration file option (‘trace.enabled = true’) generates the execution tracing file for all pipeline runs as well. The file contains task information including the submission time, start time, completion time, CPU usage, memory usage, and disk usage. Nextpie will receive the file automatically from a properly configured Nextflow pipeline via the RESTful API. The user also has the option to upload the file from Nextpie’s web-interface manually. When the manual-upload option is used, a user must provide a trace file generated by a Nextflow pipeline and pipeline details, such as the pipeline name and version. The pipeline details can either be provided via the HTML form or by uploading a text file containing those details. Similarly, a user may supply optional fields, such as a research group name and a project name, either via the form or via the same text file.

Nextpie comes with an SQLite database populated with test data. The test data allows users to explore Nextpie’s features and capabilities. The test data can be removed using flask’s command-line interface or Nextpie’s API. Detailed instructions can be found from Nextpie’s GitHub repository (https://github.com/bishwaG/Nextpie/blob/main/docs/db-clear-test-data.md).

### 3.2 Graphical user interface

Nextpie has three different graphical user interfaces (GUI): a default interface, an API interface, and an administrative interface. The default interface is behind a password protected area. It is broadly divided into two parts: one for the database and the other for the analysis. A user can upload resource usage metadata manually, generate custom plots and view, generate and export aggregated plots (Fig. 2) via the default interface. In addition, this interface allows a user to browse database records from which to perform basic analyses and generate custom plots.

Nextpie’s second interface is an API interface, a RESTful API endpoint which allows a user to interact with Nextpie using a web browser, a programming language of choice (e.g. Python or Java), or via a command-line, using curl. An API key is required for an API to function. The key can be generated from the default interface by clicking the Generate a key button from the Settings page. The administrative interface allows a power user to manage user access, view and edit database entries. This interface is accessible to power users using their same password as for the default interface.

### 3.3 Integration

Nextpie uses nf-nextpie, a Nextflow plugin to upload all relevant data to Nextpie via Nextpie’s API upon completion of a run initiated by Nextflow’s workflow.onComplete handler. The plugin is listed in Nextflow’s plugin repository index. Thus, a Nextflow pipeline automatically downloads the plugin from the plugin repository, if not available locally. However, a user must either supply the plugin name and the version (e.g. -plugins nf-nextpie@0.0.2) via Nextflow’s command-line option -plugins to use it during a single pipeline run, or add the plugin name and the version (e.g. plugins {id 'nf-nextpie@0.0.2'}) into the pipeline configuration file (nextflow.config) to use in every pipeline run. The nf-nextpie plugin includes a JSON (JavaScript Object Notation) formatted configuration file config.json, which contains the following details:

- The host IP address where Nextpie is running.
- The host port number on which Nextpie is running.
- An API key to authenticate with Nextpie.
- The name of the Nextflow variable that stores the pipe-line name.
- The name of the Nextflow variable that stores the pipe-line version.

A user can upload data to Nextpie without modifying the configuration file. However, it is recommended to avoid using the default API key for security reasons when deployed for production use. This minimal configurational change requirement provides a low threshold for users to utilize Nextpie. Nextpie’s GitHub repository contains an example Nextflow pipeline for testing. The example pipeline also serves as a template for integrating Nextpie into Nextflow pipelines.

### 3.4 Software environment

Nextpie can be run inside a variety of popular software environments. The following are the software environments that are currently supported by Nextpie:

- Python virtual environment
- Conda environment
- Guix environment (Vallet *et al.* 2022)
- Docker container

## 4 Discussion

### 4.1 Database

Nextpie enables the collection of pipeline metadata passively via an API into a centralized database. The collected data are thus not restricted to Nextpie’s user interface, but can be used more generally. The database records can be edited and updated using SQL syntax. In addition, relational database tools can be used for data manipulation, reporting and visualization. Aggregating data in this manner allows for far more time- and resource-efficient analysis than the processing of data stored in multiple files.

### 4.2 User interface

A user interface is a communication endpoint between a human and a computer or piece of software. It plays a crucial role in the user’s experience. Nextpie offers users both a GUI and a command-line interface to interact with it. Being a web-based application, a web browser is required to access Nextpie’s interface. Through the interface, a user can access the database records, aggregate them, perform basic analyses, and generate plots. In addition, the interface offers a wide range of interactive visualizations of resource usage from different viewpoints. Nextpie helps pipeline developers to gain a high-level overview of how their workflows are performing (Fig. 2A). Plots generated by Nextpie give a birds-eye view of the stability of pipeline versions by comparing failed and completed workflows. Nextpie implements an extension of the flask command-line interface that allows a power user to remove, edit and backup database records. The command-line interface provides an option for automated database backup using cron, a job scheduler in Linux operating systems.

### 4.3 Software environment

Software environments are commonly used in data science for managing software dependencies without requiring much reliance on operating system-wide library and binary installations. Usage of software environments is becoming increasingly popular in bioinformatics because software dependencies can be incapsulated in an environment to make it portable and reproducible. Nextpie can be run within a Python virtual environment, Conda environment, Guix environment, or as a Docker container. Nextpie’s Github repository contains configuration files to create the respective software environments. Creating environments from these files decreases the setup time and reduces setup complexity. Moreover, a user can deploy Nextpie instantly without configuration using the containerized Nextpie image from Docker Hub, significantly simplifying the setup process.

### 4.4 The RESTful application programming interface (API)

A RESTful API or REST API (https://www.w3.org/2001/sw/wiki/REST) is widely implemented in web applications (Golmohammadi *et al.* 2024) to communicate or exchange data with external software. It is a stateless protocol using HTTP (hypertext transfer protocol) methods. RESTful APIs are gaining traction in bioinformatics, and it is increasingly becoming the norm to make a RESTful API endpoint available to query and retrieve data from a database. For example, the European Bioinformatics Institute (EMBL-EBI) has a RESTful service called WEDL (https://www.ebi.ac.uk/ebisearch/documentation/rest-api) to facilitate easy access to biological data hosted at the institute.

RESTful API has been implemented in Nextpie to populate the database automatically using the resource usage metadata from Nextflow pipelines. The API can also retrieve data from the database. The API returns data in the JSON (JavaScript Object Notation) format, a popular data format used in the web (Neumann *et al.* 2021). JSON-formatted data are human-readable and compatible with different programming languages and frameworks. Data extracted via the API therefore gives a user the flexibility to perform custom visualizations and analyses which would otherwise not be possible via the default interface. The API endpoint has Swagger (https://swagger.io) API documentation. The documentation contains details on input data types, returned data types and HTTP response status codes. This helps the user to understand how an API works. A graphical user interface is available as part of the API documentation, giving the user the option to try the API by providing prompted inputs.

### 4.5 The nf-core pipelines

Sequencing centers and bioinformatics core units regularly run some form of automated data analysis pipeline. With the availability of nf-core (Ewels *et al.* 2020) community-written Nextflow workflows, it is becoming ever easier to deploy Nextflow based pipelines on in-house or cloud computing environments. The nf-core has 136 bioinformatics pipelines (https://nf-co.re/pipelines) available to date. New pipelines are being continuously developed, improved upon, and maintained by the bioinformatics community. Nextpie’s nf-nextpie plugin offers the possibility of seamless integration with such workflows to gain added value.

### 4.6 Existing solution

Nextflow Tower (https://tower.nf), which is now a part of Seqera Cloud (https://cloud.seqera.io), provides computational resource usage plots along with a pipeline monitoring feature for a single pipeline run. However, it still lacks a pipeline run aggregation feature, and the visualizations offered are limited to CPU usage/efficiency, memory usage/efficiency, running time, and disk input/output. Nextpie, on the other hand, provides a wide range of visualizations of aggregated data with API support. Moreover, contrary to Seqera Cloud, Nextpie is open-source and delivered as SaaP (Software as a Product). Thus, it can be deployed on a private server or a server over the Internet, depending on the need.

## 5 Conclusion

Nextpie provides improved insights in terms of computational resource usage for pipeline developers and system administrators by providing comprehensive aggregated visualizations. The wide range of deployment methods offers flexibility and, when deployed using a prebuilt Docker image, it offers a low threshold for users to try because of the zero configuration needed. While the majority of Nextflow pipelines rely on CPUs for data processing, Nextflow pipelines can equally exploit a graphics processing unit (GPU) for demanding data analysis. However, Nextflow pipelines still lack resource usage data concerning GPUs in the trace files. Because of that, visualizations covering GPU usage are missing in Nextpie as well. Moreover, Nextpie is intended primarily for facilities or research units producing genomics data on a regular basis and processing them routinely using Nextflow pipelines. This means that a researcher running a Nextflow pipeline on a small scale will likely not perceive the benefits of Nextpie.

## Supplementary Material

vbaf252_Supplementary_Data

## Data Availability

Test data are available in an SQLite database. The database file can be found from the GitHub or from Zenodo at https://doi.org/10.5281/zenodo.12732595. The test data was generated using current Nextflow pipelines such as FIMM-RNAseq (Kangas *et al.* 2022) and RNAseqVar (https://version.helsinki.fi/fimm/rnaseqvar) on real datasets. The original sample identifiers have been anonymized. The example pipeline contains FASTQ files from GEO (accession no. GSE71165). The Nextpie source code is available from the GitHub (https://github.com/bishwaG/Nextpie) under MIT license. In addition to the source code, the repository contains the version information, user manual and an example pipeline. The repository also contains the configuration files for running Nextpie in different software environments. Nextpie’s software dependency tree is created automatically during the creation of an environment. Nextpie GitHub repository is archived in Zenodo (https://doi.org/10.5281/zenodo.12732595) as well. A Docker image is available from Dockerhub (https://hub.docker.com/r/fimmtech/nextpie). The Nextflow plugin nf-nextpie is available from GitHub (https://github.com/bishwaG/nf-nextpie) and Zenodo (https://doi.org/10.5281/zenodo.15784236).
